# Dose-dependent volume loss in subcortical deep grey matter structures after cranial radiotherapy

**DOI:** 10.1016/j.ctro.2020.11.005

**Published:** 2020-11-15

**Authors:** Steven H.J. Nagtegaal, Szabolcs David, Marielle E.P. Philippens, Tom J. Snijders, Alexander Leemans, Joost J.C. Verhoeff

**Affiliations:** a Department of Radiation Oncology, University Medical Center Utrecht, HP Q 00.3.11, PO Box 85500, 3508 GA Utrecht, the Netherlands; b UMC Utrecht Brain Center, Department of Neurology & Neurosurgery, University Medical Center Utrecht, HP L 01.310, PO Box 85500, 3508 GA Utrecht, the Netherlands; c Image Sciences Institute, University Medical Center Utrecht, HP Q 00.3.11, PO Box 85500, 3508 GA Utrecht, the Netherlands

**Keywords:** CAT12, computational anatomy toolbox 12, CT, computed tomography, FWER, family-wise error rate, GM, grey matter, MRI, magnetic resonance imaging, PALM, permutation analysis of linear models, PTV, planning target volume, RT, radiotherapy, SPM, statistical parametric mapping, TFE, turbo fast echo, WBRT, whole-brain radiotherapy, Radiotherapy, Brain neoplasms, Gray matter, Amygdala, Nucleus accumbens, Caudate nucleus, Hippocampus, Globus pallidus, Putamen, Thalamus

## Abstract

**Background and purpose:**

The relation between radiotherapy (RT) dose to the brain and morphological changes in healthy tissue has seen recent increased interest. There already is evidence for changes in the cerebral cortex and white matter, as well as selected subcortical grey matter (GM) structures. We studied this relation in all deep GM structures, to help understand the aetiology of post-RT neurocognitive symptoms.

**Materials and methods:**

We selected 31 patients treated with RT for grade II-IV glioma. Pre-RT and 1 year post-RT 3D T1-weighted MRIs were automatically segmented, and the changes in volume of the following structures were assessed: amygdala, nucleus accumbens, caudate nucleus, hippocampus, globus pallidus, putamen, and thalamus. The volumetric changes were related to the mean RT dose received by each structure. Hippocampal volumes were entered into a population-based nomogram to estimate hippocampal age.

**Results:**

A significant relation between RT dose and volume loss was seen in all examined structures, except the caudate nucleus. The volume loss rates ranged from 0.16 to 1.37%/Gy, corresponding to 4.9–41.2% per 30 Gy. Hippocampal age, as derived from the nomogram, was seen to increase by a median of 11 years.

**Conclusion:**

Almost all subcortical GM structures are susceptible to radiation-induced volume loss, with higher volume loss being observed with increasing dose. Volume loss of these structures is associated with neurological deterioration, including cognitive decline, in neurodegenerative diseases. To support a causal relationship between radiation-induced deep GM loss and neurocognitive functioning in glioma patients, future studies are needed that directly correlate volumetrics to clinical outcomes.

## Highlights

• Subcortical grey matter is susceptible to dose-dependent volume loss after RT.
• Hippocampal age increases 1 year after radiotherapy, by a median of 11 years.
• We may need to reconsider current sparing strategies in RT for brain tumours.
• Future studies should examine the impact of deep GM volume loss on cognition.

## 1 Introduction

Irradiation of healthy brain tissue can lead to anatomical and functional deficits, a phenomenon known as radiation-induced brain injury. This can lead to a variety of symptoms, with especially cognitive and executional impairments leading to a marked decrease in the patient’s quality of life after radiation therapy (RT) [1], [2].

With the advent of high-resolution brain imaging, the interest in morphological changes after RT has increased. The cerebral cortex has been shown to be susceptible to radiation-induced thinning, especially in areas associated with cognitive functioning [3], [4], [5], [6]. Thinning rates are found to be dose-dependent, meaning that a higher dose leads to a further diminished cortex. Similarly, diffusion tensor imaging has shown that white matter shows dose-dependent changes in several metrics after RT [7]. Finally, two grey matter structures, the hippocampus [8], [9] and the amygdala [10], show susceptibility to radiation damage, again with higher volume changes with increasing dose. Furthermore, the dose to the hippocampus has been shown to negatively affect neurocognitive outcome after RT [11].

Less is known about the susceptibility to radiation damage of other subcortical grey matter structures, such as the nucleus accumbens, caudate nucleus, globus pallidus, putamen, and thalamus. Atrophy of these deep GM structures is associated with impaired cognitive function in patients with degenerative brain diseases as well as healthy ageing [12], [13], [14]. This relation is most pronounced in Alzheimer’s disease, with the volume of all mentioned structures, with the exception of globus pallidus, being associated with cognitive impairment [15], [16], [17]. Globus pallidus volume in its turn is associated with cognitive outcomes in Huntington’s disease and age-related cognitive impairments [18], [19].

Volume changes in these structures are associated with cognitive outcomes, and the cause of post-RT cognitive decline needs to be elucidated. Therefore, we examined the relation between post-RT subcortical GM volume changes and RT dose.

## 2 Methods

### 2.1 Patient selection and data collection

Patients who were treated with photon intensity-modulated radiation therapy for newly discovered grade II-IV glioma at the department of Radiation Oncology in 2016 and 2017 were retrospectively identified. Criteria for inclusion were: treatment planning CT and MRI present, with isotropic high resolution; survival > 270 days after start of RT; and availability of at least 1 follow-up MRI between 270 days and 360 days after start of RT, and with isotropic high resolution. Patients were excluded in case of tumour progression or recurrence between baseline and follow-up. Clinical MRI and CT scans made for RT treatment planning, all follow-up MRIs, and clinical and demographic characteristics were extracted from patient records. The need for informed consent for this retrospective study was waived by our institutional review board (#18/274).

### 2.2 Image acquisition

For every patient the pre-RT CT and MRI were collected, as well as all available follow-up MRIs. RT planning CT scans were acquired on a Brilliance Big bore scanner (Philips Medical Systems, Best, The Netherlands), with a tube potential of 120 kVp, with a matrix size of 512 × 512 and 0.65 × 0.65 × 3.0 mm voxel size. MR images were acquired on a 3 T Philips Ingenia scanner (Philips Healthcare, Best, The Netherlands) as part of routine clinical care. T1-weighted MR images were acquired with a 3D turbo field echo (TFE) sequence without gadolinium enhancement with the following parameters: TR = 8.1 ms, TE = 3.7 ms, flip angle = 8°, matrix: 207 × 289 × 213, and a reconstructed voxel resolution of 1 × 0.96 × 0.96 mm.

### 2.3 Image processing

**Fig. 1:**
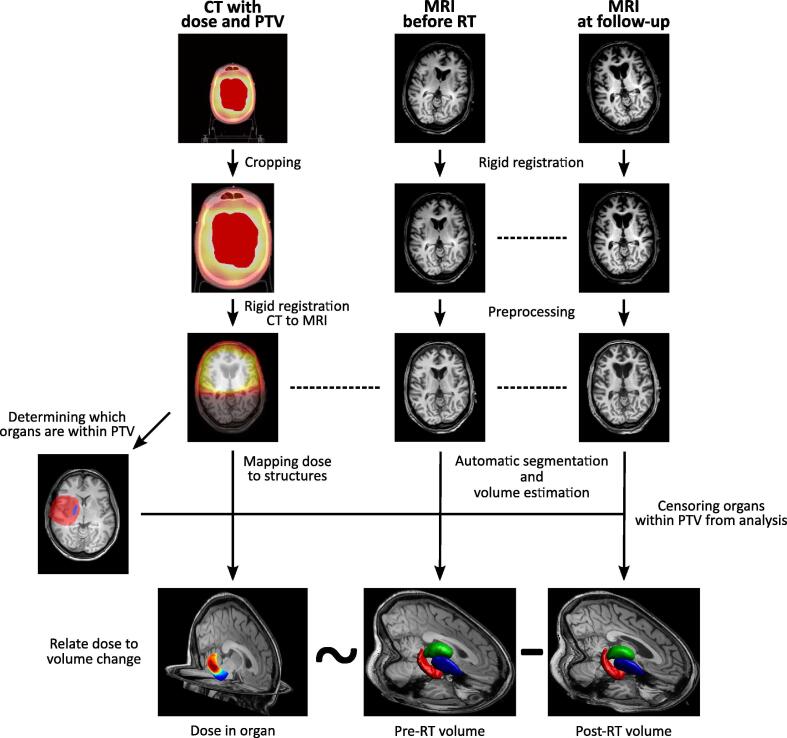
Pipeline of image processing. Left column: dose (colour gradient) and PTV (red shading) are extracted from CT. Middle and right column: organ volume is estimated from processed MRIs before RT and at follow-up. CT and MRI are registered to each other (dotted lines), and organs within PTV are censored from analysis. Finally applied dose is related to the change in organ volume. Note that the images used are used illustratively, and do not represent a single case. (For interpretation of the references to colour in this figure legend, the reader is referred to the web version of this article.)

A graphical overview of the image processing pipeline is shown in Fig. 1**.** All imaging data was processed with Statistical Parametric Mapping (SPM12, v7487) [20], Computational Anatomy Toolbox (CAT12.6 r1450) [21], and in-house algorithms developed in MATLAB (Mathworks, Natick, Massachusetts, USA). Image processing was done in concordance to our own previously published criteria [3], amended for the current research question. More detailed image processing methods can be found in our previous work [4]**.**

In brief, the cropped CT image with the associated dose and planning target volume (PTV) maps were registered to the T1 MR images, resulting in the CT image and the MRIs being in the same space. Next, the rigidly coregistered T1s were processed with CAT12′s segmentation pipeline.

**Fig. 2:**
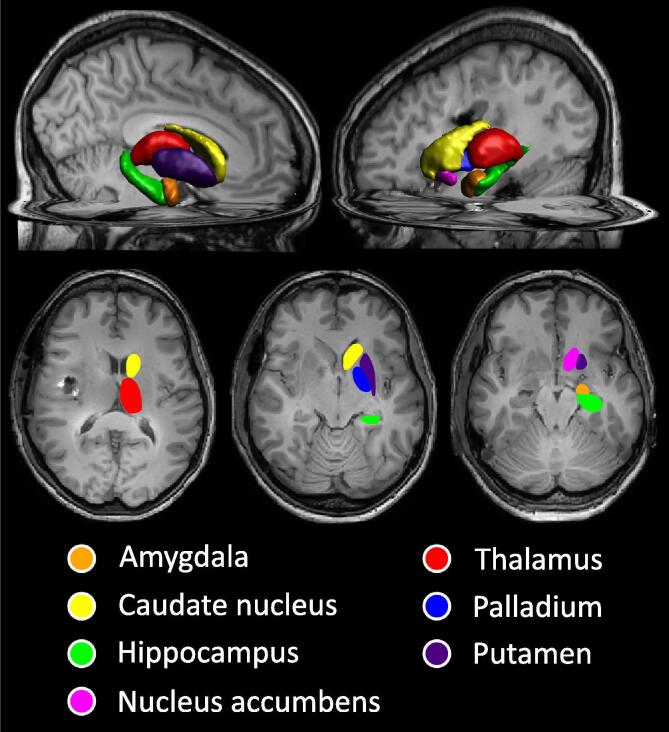
A 3D rendering and axial MR images showing the subcortical grey matter structures being analysed.

Deep GM structure volumes were estimated with CAT12 using the fully automated volume estimation method using the labels from the Neuromorphometrics atlas (Neuromorphometrics Inc., Somerville, Massachusetts, USA). The following structures were examined: amygdala, nucleus accumbens, caudate nucleus, hippocampus, globus pallidus, putamen, and thalamus. Fig. 2 shows the anatomical location of the structures on axial T1 MRI, as well as in a 3D rendering. This resulted in 14 volumes per patient, as the GM volumes for the left and right hemisphere were separately estimated.

For the primary analysis, we analysed the difference in volume between baseline and 1 year follow-up. The latter was defined as the time point closest to 360 days after start of RT for which an MRI was available.

The within-subject difference in deep GM volume was calculated by subtracting the baseline and the 1-year follow-up volume. In every subject the deep GM organs included in the PTV were censored from analysis, to avoid spurious volume-dose relations originating from segmentation errors due to damage around the tumour [22]. If the residual damage (e.g. oedema, surgical scarring, tumour bed) extended beyond the PTV in either the baseline or the follow-up images, then the affected subject was removed from the analysis.

### 2.4 Statistical analysis

We correlated the change in deep GM volume after 1 year with the mean dose received by each subcortical structure. Statistical comparison of deep GM volume change and dose correlation was carried out with a permutation test with 10,000 iterations performed with the permutation analysis of linear models (PALM) toolbox in Matlab [23], [24], [25]. Significance of a correlation was set at p_corr_ < 0.05, with use of family-wise error rate (FWER) adjustment to correct for multiple comparisons. All further presented p-values are FWER-corrected. Age at the time of the diagnosis and sex of the patients were included as nuisance regressors.

To assess whether administration of chemotherapy has an effect on the relation between dose and volume, a sensitivity analysis was performed in which chemotherapy was added as a covariate to the permutation test, again with FWER-adjustment.

### 2.5 Hippocampal nomograms

To put volumetric changes of the hippocampus into context, the pre-RT and post-RT volumes were entered into a nomogram of hippocampal volume across age groups [26], [27]. This nomogram is based on MRI data from 19,700 healthy participants from the UK Biobank. We did this in two ways: 1) the patients’ new “hippocampal age” was determined based on its volume after RT and the percentile in the nomogram at baseline; this was then compared to the actual age at baseline, and 2) we assessed whether there was a change in a patient’s hippocampal volume percentile within the population between the pre-RT and post-RT scans. For this analysis we used not only the 1-year post-RT MRI, but all available follow-up MRIs. Due to the age range of the nomograms, only patients aged 52 to 72 could be entered into the nomogram. When hippocampal volumes at follow-up were below the limits of the nomogram, the hippocampal age was set at the maximum age that could be derived from the reference dataset (i.e. 72).

## 3 Results

### 3.1 Participants

Of all the patients treated with RT for glioma in 2016 and 2017, thirty-one patients were eligible for inclusion in the current analysis. A flow-chart of study inclusion is shown in Supplementary Fig. 1. Extensive damage outside the censored PTV area on baseline MRI meant exclusion of one case. Baseline characteristics are shown in Table 1**.** Median follow-up time of the used MRI assessments was 319 days, with a range of 270–360.

**Table 1:** Baseline characteristics of included patients.

	N (total n = 31)
**Age (mean; SD)**	50 (±15)
**Sex**	
Male	19 (61.3%)
Female	12 (38.7%)
**WHO grade**	
II	12 (38.7%)
III	6 (19.4%)
IV	13 (41.9%)
**Tumour type**	
Astrocytoma, IDH-mutant	13 (41.9%)
Astrocytoma, IDH-wildtype	3 (9.6%)
Glioblastoma, IDH-wildtype	9 (29.0%)
Other	6 (19.6%)
**Prescribed dose**	
28 × 1.8 Gy = 50.4 Gy	11 (35.5%)
30 × 1.8 Gy = 54 Gy	2 (6.5%)
30 × 2.0 Gy = 60 Gy	18 (58.1%)
**Concurrent or sequential systemic therapy**	
None	5 (16.1%)
Temozolomide	21 (67.7%)
PCV	4 (12.9%)

PCV = procarbazine, lomustine and vincristine.

### 3.2 Subcortical GM volume

**Fig. 3:**
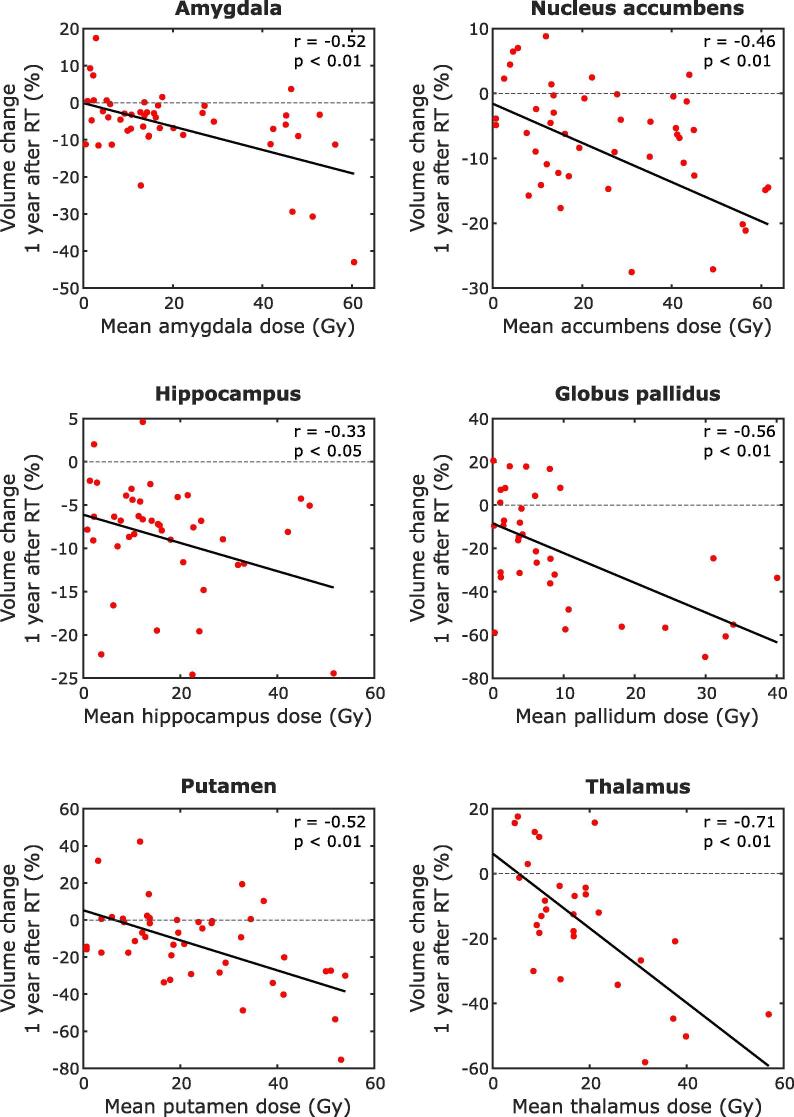
Scatterplots showing the relation between mean RT dose and volume loss in the structures where this was significant, with fitted linear regression lines.

Significant dose-dependent volume loss 1 year after RT was observed in all examined structures, except for caudate nucleus. Rates of volume loss vary from 0.16 to 1.37% per Gy (corresponding to 4.9% and 41.2% per 30 Gy), and are shown for all structures in Table 2. Scatterplots of the organs in which a significant relation between RT dose and volume loss was seen are shown in Fig. 3. Doses received per structure are shown in Supplementary Table 2.

**Table 2:** Dose-dependent changes in volumes of subcortical GM structures.

	N of structures	N of patients	Volume loss rate (%/Gy)	Volume loss rate (%/30 Gy)	95% confidence interval	p*
Amygdala	48	29	0.30	9.0	0.15–0.45	**<0.01**
Caudate nucleus	38	22	0.10	3.0	−0.22–0.42	0.74
Globus pallidus	35	22	1.37	41.2	0.68–2.07	**<0.01**
Hippocampus	42	28	0.16	4.9	0.02–0.31	**0.03**
Nucleus accumbens	45	25	0.32	9.5	0.13–0.50	**<0.01**
Putamen	44	29	0.81	24.3	0.42–1.20	**<0.01**
Thalamus	29	19	1.15	34.5	0.74–1.56	**<0.01**

* Corrected for multiple testing.

The sensitivity analysis done to assess the effect of chemotherapy on this relation is shown in Supplementary Table 2. It did not result in a change in direction or effect size of the results, and chemotherapy administration did not significantly affect GM volume.

### 3.3 Hippocampal volume nomograms

**Fig. 4:**
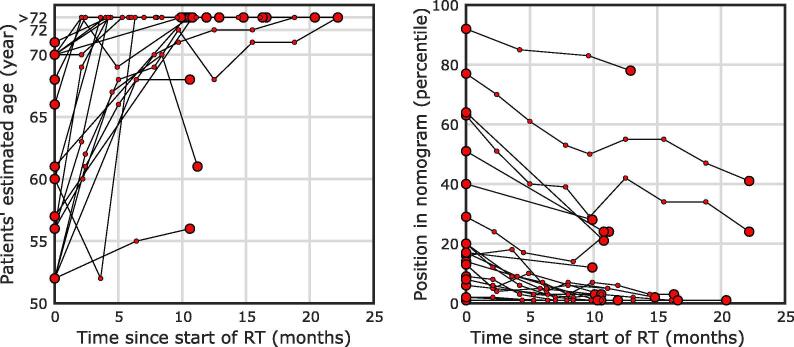
Change in patients’ hippocampal age (left) and position within the nomogram (right) based on the UK Biobank [26], estimated using all available clinical MRIs. Hippocampal age saturates at the top of the graph because age within the nomogram has a maximum of 72. Large points denote first and last available MRIs, small points those in between.

In this cohort 22 patients were within the age range of 52 to 72, and thus were entered into the nomograms from the UK Biobank, which are shown in Fig. 4. All patients show an overall increase in hippocampal age, with a median increase of eleven years (range 2–20 years). Accordingly, the percentile within the nomogram dropped for all patients, meaning that their hippocampal volume shows a decrease compared to their peers of the same age.

## 4 Discussion

When analysing structures outside of the treated PTVs, we have found that all subcortical deep grey matter structures, with the exception of caudate nucleus, show dose-dependent volume loss 1 year after RT. For the hippocampus, we have also shown that, based on data from the normal population, its volume-based age increases with up to twenty years during the year post-radiation.

The amygdala and hippocampus have shown to be susceptible to radiation damage in previous studies. Seibert et al. [8] studied MRIs before and one year after RT of 52 patients with primary brain tumours. Automatic segmentation of the hippocampus was followed by relating the difference in volume to the mean dose received. They found a significant correlation, with a Pearson correlation coefficient of −0.24. Furthermore, they found that the hippocampus showed significant volume loss after high-dose RT (defined as > 40 Gy). This in contrast to low-dose (<10 Gy), which showed no significant relation with post-RT volume. A linear mixed-effects model resulted in a volume loss rate of 0.13%/Gy, which is similar to our observed loss rate of 0.16%/Gy.

The volumetric changes in the amygdala after RT have been studied by Huynh-Le et al. [10] in the same cohort of 52 patients. A significant Pearson correlation of −0.28 was found for amygdala volume and mean dose, with a volume loss rate of 0.17%/Gy. The difference to our findings of a correlation coefficient of −0.52 and volume loss rate of 0.30%/Gy could be explained by the difference in censoring method. They censored amygdalae manually when a visual inspection deemed the segmentation to be poor, whereas we censored more strictly by censoring any organ within the PTV. This meant that they had more data points within higher dose regions, which could have led to a different slope and correlation coefficient.

Finally, a link between post-RT hippocampal volume and neurocognitive outcomes was found in primary brain tumours by Tringale et al. [9]. They found that, in addition to diffusion biomarkers, a smaller right hippocampal volume was associated with poorer visuospatial memory performance in the 12 months after RT.

A link between the volumes of these structures and cognitive outcomes has been thoroughly examined in other brain diseases. Particularly in Alzheimer’s disease, available evidence points towards a strong relation between subcortical GM structures and cognitive impairments for each of the structures we studied, except for globus pallidus [15], [16], [17]. Furthermore, cognitive impairments in Parkinson’s disease [28], [29], multiple sclerosis [30], Huntington disease [18], as well as in normal ageing [13], have been linked with the volume of at least one of the subcortical GM structures. Supplementary Table 3 gives an overview of some available literature per GM structure.

The effect of hippocampal dose and neurocognitive outcomes was first shown by Gondi et al. [11]. They showed that radiation dose of > 7.3 Gy to 40% of the bilateral hippocampi was associated with an impairment in the Wechsler Memory Scale-III Word List delayed recall test. The same group conducted phase II and phase III trials, the latter studying the effect of whole brain radiotherapy (WBRT) with or without hippocampal avoidance [31], [32]. They found hippocampal avoidance WBRT, in combination with the N-methyl-D-aspartate inhibitor memantine, preserves cognitive function while maintaining the same overall and progression-free survival.

In this study the investigation of caudate nucleus volume change in relation to the local dose was inconclusive. One explanation could be that the quality of the segmentation is region dependent. While generally the segmentations of SPM/CAT12 are highly reproducible [33], [34], [35], among the investigated regions caudate requires the largest sample size to achieve the same statistical power compared to other regions [36]. The caudate nucleus shares a relatively large interface with the ventricles (Fig. 2), making it highly susceptible to partial voluming artefacts, which may lead to errors in segmentation.

Our results challenge us to reconsider the currently used sparing strategies in radiation treatment of brain tumours. Presently, hippocampal sparing RT has been adopted in several institutions. However, sparing the dose in the hippocampus leads to higher doses in surrounding cerebral tissues, which we have shown to be susceptible to radiation-induced damage as well [37]. Future research has to focus on the relation between clinical outcomes (including cognitive and motor function) and morphologic changes, both in the entire brain and in selected structures. This way we can conclusively say which structures should be avoided in RT planning to prevent radiation-induced damage. Specific sparing of healthy brain is possible with novel techniques such as proton therapy and VMAT. Especially in intensity-modulated proton therapy, doses to organs at risk can be optimally reduced [38], meaning this technique may prove useful in preventing post-RT cognitive decline. Additionally, the relative biological effectiveness (RBE) for several substructures is still unknown and may impact the effect of radiation. This could lead to improved cognition and quality of life in patients undergoing treatment for brain tumours.

There are several limitations to this study. Firstly, we have a relatively limited sample size due to the strenuous inclusion criteria. However, these criteria ensure that the quality of the imaging used in analysis are optimal, meaning more reliable and replicable results. The censoring of the PTV also means exclusion of several subcortical GM structures, but this again is to ensure reliable automated measurements. Attempts could have been made to manually delineate these structures, but this would have added an extra variable to the dose/volume relation (manual vs automatic segmentation in high and lower dose, respectively). Additionally, it is unclear which method gives the most reliable results in patients who underwent RT. In previous works [39], [40], [41], the automated segmentation method used in the current study was rigorously compared to manual segmentation of subjects' T1 MRI data as well as brain phantoms representing a wide range of settings (noise, artefacts, etc.). It was found that CAT12 performed on a comparable level versus manual segmentation in healthy subjects as well as patients with ischemic stroke or temporal lobe epilepsy, suggesting it is reliable for segmentation in RT patients.

Another consideration is that susceptibility to radiation-induced volume loss of brain tissue might differ between patients. As each patient provides multiple organs for examination, this could have impacted the found results. We could not correct for this in the current study, as our sample size limited our ability to apply multilevel modelling of the dose/volume relation. For similar reasons, we were unable to properly model the longitudinal changes over time, but looked only at the change 1 year after RT.

Secondly, the patients in our cohort did not only undergo RT. Many also received chemotherapy, which has been linked to cerebral changes in non-neurological malignancies [42], [43]. Our analysis focussed on the association between RT dose and volume, and by relating these two factors to each other we have limited the effect of chemotherapy as much as possible. Furthermore, a sensitivity analysis including chemotherapy in the model did not give different results, suggesting its role is limited.

Finally, the absence of neurocognitive outcome data from this cohort means we cannot yet give clinical recommendations on which organs to spare.

In conclusion, subcortical grey matter structures show susceptibility to dose-dependent volume loss after radiotherapy. If neurocognitive outcomes are related to this phenomenon, current RT strategies need to be revised, in order to improve patients’ quality of life after cancer treatment.

## Declaration of Competing Interest

The authors declare that they have no known competing financial interests or personal relationships that could have appeared to influence the work reported in this paper.
