# Supplementary material for: Dose-dependent volume loss in subcortical deep grey matter structures after cranial radiotherapy
Source: Clin Transl Radiat Oncol. 2020 Nov 15;26:35–41. doi: 10.1016/j.ctro.2020.11.005 (PMC7691672; doi:10.1016/j.ctro.2020.11.005)
Supplement: Supplementary data 1 [file mmc1.pptx]

## Slide 1
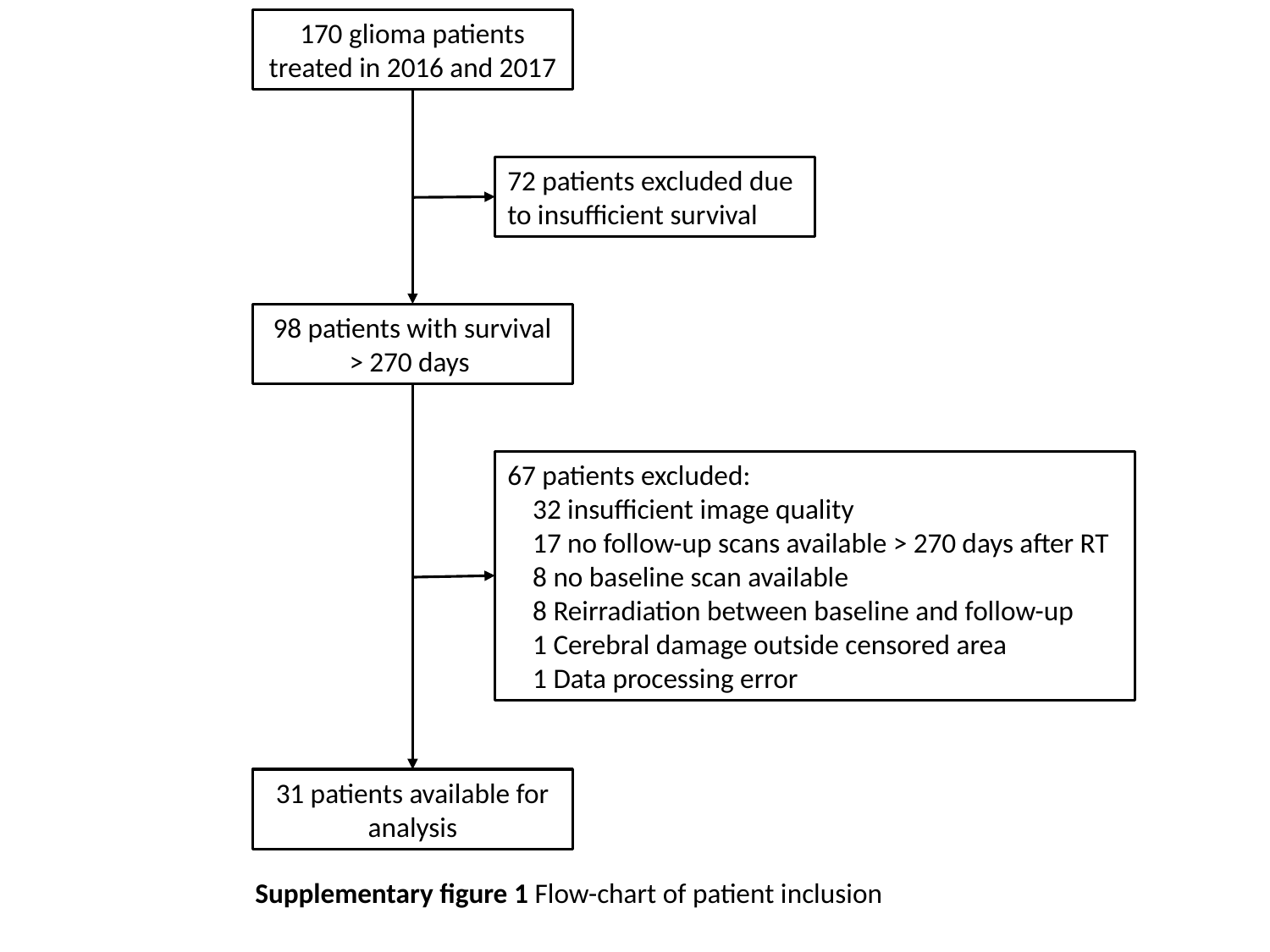

170 glioma patients treated in 2016 and 2017
72 patients excluded due to insufficient survival
98 patients with survival > 270 days
67 patients excluded:
32 insufficient image quality
17 no follow-up scans available > 270 days after RT
8 no baseline scan available
8 Reirradiation between baseline and follow-up
1 Cerebral damage outside censored area
1 Data processing error
31 patients available for analysis
Supplementary figure 1 Flow-chart of patient inclusion
