# Supplementary material for: Dose-dependent volume loss in subcortical deep grey matter structures after cranial radiotherapy
Source: Clin Transl Radiat Oncol. 2020 Nov 15;26:35–41. doi: 10.1016/j.ctro.2020.11.005 (PMC7691672; doi:10.1016/j.ctro.2020.11.005)
Supplement: Supplementary data 2 [file mmc2.docx]

| **Supplementary table 1** Dose received by each studied GM structure | | | |
| --- | --- | --- | --- |
|  | **D_mean_ (Gy)** | **D_min_ (Gy)** | **D_max_ (Gy)** |
| Amygdala | 19.7 | 0.5 | 60.5 |
| Caudate nucleus | 17.6 | 0.4 | 37.0 |
| Globus pallidus | 9.5 | 0.1 | 40.1 |
| Hippocampus | 16.8 | 0.8 | 51.6 |
| Nucleus accumbens | 25.4 | 0.7 | 61.5 |
| Putamen | 22.9 | 0.7 | 54.0 |
| Thalamus | 18.4 | 4.5 | 56.9 |
