# Supplementary material for: Dose-dependent volume loss in subcortical deep grey matter structures after cranial radiotherapy
Source: Clin Transl Radiat Oncol. 2020 Nov 15;26:35–41. doi: 10.1016/j.ctro.2020.11.005 (PMC7691672; doi:10.1016/j.ctro.2020.11.005)
Supplement: Supplementary data 3 [file mmc3.docx]

| **Supplementary table 2** Dose-dependent changes in volumes of subcortical GM structures, corrected for chemotherapy | | | | | |
| --- | --- | --- | --- | --- | --- |
|  | **n** | **Volume loss rate (%/Gy)** | **Volume loss rate (%/30Gy)** | **95% confidence interval** | **p^*^** |
| Amygdala | 48 | 0.36 | 10.7 | 0.20 – 0.51 | **<0.01** |
| Caudate nucleus | 38 | -0.11 | -3.2 | -0.43 – 0.21 | 0.94 |
| Globus pallidus | 35 | 1.31 | 39.2 | 0.64 – 1.97 | **<0.01** |
| Hippocampus | 42 | 0.17 | 5.1 | 0.03 – 0.32 | **0.02** |
| Nucleus accumbens | 45 | 0.32 | 9.5 | 0.13 – 0.50 | **<0.01** |
| Putamen | 44 | 0.83 | 24.8 | 0.44 – 1.21 | **<0.01** |
| Thalamus | 29 | 1.13 | 34 | 0.71 – 1.56 | **<0.01** |
| *corrected for multiple testing | | | | | |
