# Supplementary material for: Dose-dependent volume loss in subcortical deep grey matter structures after cranial radiotherapy
Source: Clin Transl Radiat Oncol. 2020 Nov 15;26:35–41. doi: 10.1016/j.ctro.2020.11.005 (PMC7691672; doi:10.1016/j.ctro.2020.11.005)
Supplement: Supplementary data 4 [file mmc4.docx]

| **Supplementary table 3** Examples of previously reported links between cognitive outcomes and subcortical GM volume | | | | | |
| --- | --- | --- | --- | --- | --- |
|  | **Ageing** | **Parkinson’s** | **Alzheimer’s** | **MS** | **Huntington’s** |
| **Amygdala** | [1] | [2] | [3] |  |  |
| **Nucleus accumbens** |  | [2] | [3], [4] |  |  |
| **Caudate nucleus** |  |  | [4] |  | [5] |
| **Hippocampus** | [1], [6] |  | [3], [4] | [7] |  |
| **Globus pallidus** | [8] |  |  |  | [5] |
| **Putamen** |  | [9] | [4], [10] | [7] | [5] |
| **Thalamus** | [11] |  | [4], [10] | [7], [12] |  |

**References**

[1] D. Zanchi, P. Giannakopoulos, S. Borgwardt, C. Rodriguez, and S. Haller, “Hippocampal and Amygdala Gray Matter Loss in Elderly Controls with Subtle Cognitive Decline,” *Front. Aging Neurosci.*, vol. 9, Mar. 2017.

[2] A. Hanganu *et al.*, “Mild cognitive impairment is linked with faster rate of cortical thinning in patients with Parkinson’s disease longitudinally.,” *Brain*, vol. 137, no. Pt 4, pp. 1120–9, Apr. 2014.

[3] X. Nie *et al.*, “Subregional Structural Alterations in Hippocampus and Nucleus Accumbens Correlate with the Clinical Impairment in Patients with Alzheimer’s Disease Clinical Spectrum: Parallel Combining Volume and Vertex-Based Approach.,” *Front. Neurol.*, vol. 8, p. 399, 2017.

[4] H.-A. Yi *et al.*, “Relation between subcortical grey matter atrophy and conversion from mild cognitive impairment to Alzheimer’s disease,” *J. Neurol. Neurosurg. Psychiatry*, vol. 87, no. 4, pp. 425–432, Apr. 2016.

[5] E. H. Aylward *et al.*, “Regional atrophy associated with cognitive and motor function in prodromal Huntington disease.,” *J. Huntingtons. Dis.*, vol. 2, no. 4, pp. 477–89, 2013.

[6] A. O’Shea, R. A. Cohen, E. C. Porges, N. R. Nissim, and A. J. Woods, “Cognitive Aging and the Hippocampus in Older Adults,” *Front. Aging Neurosci.*, vol. 8, Dec. 2016.

[7] M. A. Rocca *et al.*, “Clinical and imaging assessment of cognitive dysfunction in multiple sclerosis.,” *Lancet. Neurol.*, vol. 14, no. 3, pp. 302–17, Mar. 2015.

[8] M. C. Valdés Hernández *et al.*, “The striatum, the hippocampus, and short-term memory binding: Volumetric analysis of the subcortical grey matter’s role in mild cognitive impairment,” *NeuroImage Clin.*, vol. 25, p. 102158, 2020.

[9] D. Hünerli, D. D. Emek-Savaş, B. Çavuşoğlu, B. Dönmez Çolakoğlu, E. Ada, and G. G. Yener, “Mild cognitive impairment in Parkinson’s disease is associated with decreased P300 amplitude and reduced putamen volume,” *Clin. Neurophysiol.*, vol. 130, no. 8, pp. 1208–1217, Aug. 2019.

[10] L. W. de Jong *et al.*, “Strongly reduced volumes of putamen and thalamus in Alzheimer’s disease: an MRI study.,” *Brain*, vol. 131, no. Pt 12, pp. 3277–85, Dec. 2008.

[11] E. J. Hughes *et al.*, “Regional changes in thalamic shape and volume with increasing age.,” *Neuroimage*, vol. 63, no. 3, pp. 1134–42, Nov. 2012.

[12] J. I. Rojas *et al.*, “Thalamus volume change and cognitive impairment in early relapsing-remitting multiple sclerosis patients.,” *Neuroradiol. J.*, vol. 31, no. 4, pp. 350–355, Aug. 2018.
